# Knowledge Graph–Enhanced Deep Learning Model (H-SYSTEM) for Hypertensive Intracerebral Hemorrhage: Model Development and Validation

**DOI:** 10.2196/66055

**Published:** 2025-06-12

**Authors:** Yulong Xia, Jie Li, Bo Deng, Qilin Huang, Fenglin Cai, Yanfeng Xie, Xiaochuan Sun, Quanhong Shi, Wei Dan, Yan Zhan, Li Jiang

**Affiliations:** 1 Department of Neurosurgery, The First Affiliated Hospital of Chongqing Medical University, No. 1 Youyi Road, Yuanjiagang, Yuzhong District, Chongqing, China, 86 13638354200; 2 School of Computer Science and Engineering, Chongqing University of Science and Technology, Chongqing, China

**Keywords:** hypertensive intracerebral hemorrhage, artificial intelligence, knowledge graph, medical domain knowledge graph, deep learning–based model, electronic medical record, explainability, black box

## Abstract

**Background:**

Although much progress has been made in artificial intelligence (AI), several challenges remain substantial obstacles to the development and translation of AI systems into clinical practice. Even large language models, which show excellent performance on various tasks, have progressed slowly in clinical practice tasks. Providing precise and explainable treatment plans with personalized details remains a big challenge for AI systems due to both the highly specialized medical knowledge required and patients’ complicated conditions.

**Objective:**

This study aimed to develop an explainable and efficient decision support system named H-SYSTEM to assist neurosurgeons in diagnosing and treating patients with hypertensive intracerebral hemorrhage. The system was designed to address the limitations of existing AI systems by integrating a medical domain knowledge graph to enhance decision-making accuracy and explainability.

**Methods:**

The H-SYSTEM consists of 3 main modules: the key named entity recognition (NER) module, the semantic analysis and representation module, and the reasoning module. Furthermore, we constructed a medical domain knowledge graph for hypertensive intracerebral hemorrhage, named HKG, which served as an external knowledge brain of the H-SYSTEM to enhance its text recognition and automated decision-making capability. The HKG was exploited to guide the training of the semantic analysis and representation module and reasoning module, which makes the output of the H-SYSTEM more explainable.

To assess the performance of the H-SYSTEM, we compared it with doctors and different large language models.

**Results:**

The outputs based on HKG showed reliable performance as compared with neurosurgical doctors, with an overall accuracy of 94.87%. The bidirectional encoder representations from transformers, inflated dilated convolutional neural network, bidirectional long short-term memory, and conditional random fields (BERT-IDCNN-BiLSTM-CRF) model was used as the key NER module of the H-SYSTEM due to its fast convergence and efficient extraction of key named entities, achieved the highest performance among 7 key NER models (precision=92.03, recall=90.22, and *F*_1_-score=91.11), significantly outperforming the others. The H-SYSTEM achieved an overall accuracy of 91.74% in treatment plans, showing significant consistency with the gold standard (*P*<.05), with diagnostic measures achieving 88.18% accuracy, 97.03% area under the curve (AUC), and a κ of 0.874; surgical therapy achieving 98.53% accuracy, 98.53% AUC, and a κ of 0.971; and rescue therapies achieving 89.50% accuracy, 94.67% AUC, and a κ of 0.923 (all *P*<.05). Furthermore, the H-SYSTEM showed high reliability and efficiency when compared to doctors and ChatGPT, achieving statistically higher accuracy (95.26% vs 91.48%, *P*<.05). Additionally, the H-SYSTEM achieved a total accuracy of 92.22% (ranging from 91.14% to 95.35%) in treatment plans for 605 additional patients from 6 different medical centers.

**Conclusions:**

The H-SYSTEM showed significantly high efficiency and generalization capacity in processing electronic medical records, and it provided explainable and elaborate treatment plans. Therefore, it has the potential to provide neurosurgeons with rapid and reliable decision support, especially in emergency conditions. The knowledge graph–enhanced deep-learning model exhibited excellent performance in the clinical practice tasks.

## Introduction

Acute spontaneous (nontraumatic) intracerebral hemorrhage (ICH) is one of the leading causes of death and disability worldwide, posing a massive burden on health care systems [1-3]. Hypertensive intracerebral hemorrhage (HICH) is one of the most serious types of ICH and affects approximately 2 million people worldwide each year [4-7]. Timely diagnosis and treatment are of critical clinical importance for patients with HICH, as nearly half of the resulting mortality occurs within the first 24 hours [8-10]. Thus, the development of methods that provide rapid and accurate diagnosis and treatment could significantly improve the prognosis of patients with HICH.

Artificial intelligence (AI) has lately made significant advances in the interpretation of sensory information, allowing machines to automatically analyze and comprehend complicated facts [11-16]. Although AI models like large language models (LLMs) have shown excellent performance in some general medical question–answering tasks, they do not perform so well in the clinical practice tasks [17-22]. Furthermore, the need for large, well-annotated data and the black box problem remain the two main challenges to the development and translation of medical AI systems into clinical practice [23]. Developing precise treatment plans with individualized details for patients with HICH still remains a challenge for AI systems due to not only the highly abstracted and professional medical knowledge and terminology, but also the complexity of the patient’s condition.

Aiming to address the above challenges and provide substantial supports for neurosurgeons in the clinical diagnosis and treatment of HICH cases, we developed an automatic decision support system called H-SYSTEM based on multiple-center data. We also enhanced the ability of deep learning models by constructing a specialized knowledge graph that also increases the explainability of the decision-making process.

## Methods

### Ethical Considerations

All study procedures were approved by the ethical committees of all the medical centers mentioned in this study. The patients involved in the study all signed informed consent forms before admission. All data involved in this study have been anonymized or deidentified. The case data involved in this study are part of a retrospective study. All participants received professional medical treatment at the time, and no additional compensation was provided.

### Study Design and Participants

Following ethical committees’ approval, electronic medical record (EMR) data from 15 medical centers were enrolled into the neurosurgery EMR database, HICH-IT, which was posted on GitHub [24]. More than 8000 HICH cases were extracted from this database and divided into training, validation, and testing sets, respectively. The inclusion criteria for cases were as follows: (1) patients diagnosed with spontaneous cerebral hemorrhage in compliance with the latest stroke guidelines and (2) patients aged 10-80 years old. Patients with an incomplete medical history or physical examination or without head computed tomography (CT) results were excluded.

### Development of the H-SYSTEM

#### Input and Output of the H-SYSTEM: EMR Acquisition and Preprocessing

Basic demographic information, chief complaint, history of current and past illnesses, physical examination, head CT results, and other clinical variables that would be available to doctors and AI experts were abstracted from the HICH-IT. The narrative clinical notes retrieved from the EMR database needed to undergo a series of text preprocessing steps before natural language processing techniques could be applied. These steps included using the spell checker to correct misspelled words, extending abbreviations and acronyms, and tokenization. Then, the key named entities were annotated (Multimedia Appendix 1). The first category of key named entities included the clinical and CT manifestation, such as the main complaints, vital signs, Glasgow Coma Score, abnormal pupillary sizes and reflexes, location, and nature of the hematoma. The second category included quantitative parameters of the hematoma, such as the volume of the hematoma, displacement of midline brain structures, and compression of the lateral ventricles. After performing these preprocessing steps, we finally attained a clean corpus and labels for data analysis.

#### Treatment Plans Output by the H-SYSTEM

**Figure 1.**
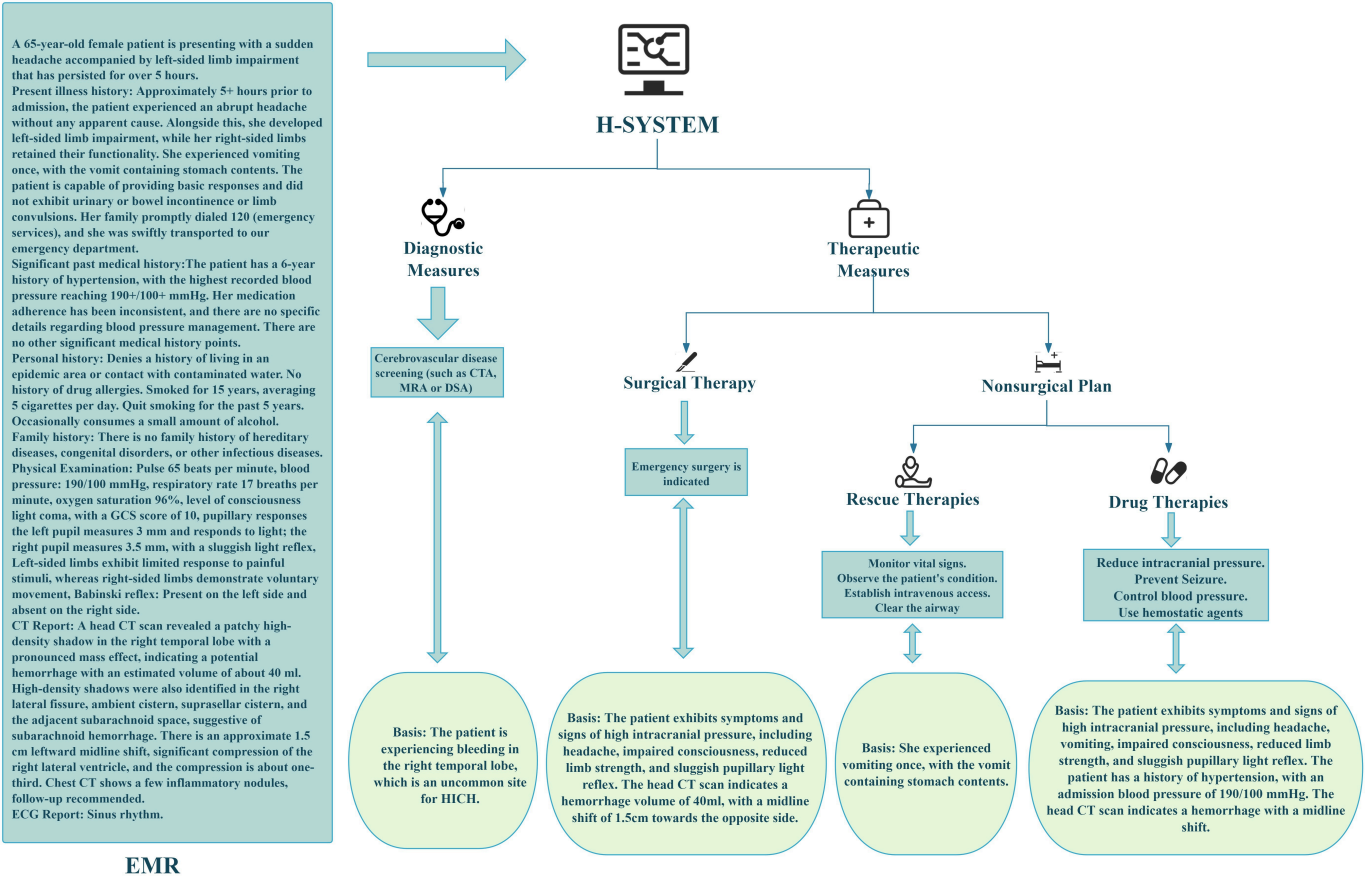
An example of the output results of the H-SYSTEM. CT: computed tomography; CTA: computed tomography angiography; DSA: digital subtraction angiography; GCS: Glasgow Coma Score; HICH: hypertensive intracerebral hemorrhage; MRA: magnetic resonance angiography.

The treatment plans output by the H-SYSTEM were divided into diagnostic and therapeutic measures. To achieve explainability, the H-SYSTEM not only outputs the treatment plans but also provides the basis of outputting the decisions, making the internal working and decision-making process explainable (Figure 1). The detailed treatment plans output by the H-SYSTEM are displayed in Multimedia Appendix 2.

#### Diagnostic Measures

The purpose of the diagnostic measures was to provide more information and further evidence when the clinical manifestation and plain CT report in the EMR were not enough to make an accurate evaluation and determine proper treatment plans. The diagnostic measures included cerebrovascular examinations (such as computed tomography angiography, magnetic resonance angiography, and digital subtraction angiography), multimodal magnetic resonance imaging (such as plain and contrast-enhanced T1, T2, T2-FLAIR), and coagulation function.

#### Therapeutic Measures

The therapeutic measures were divided into surgical therapy and nonsurgical therapy. The nonsurgical therapy was more complicated and was further divided into rescue therapies and drug therapies. The rescue therapies consisted of monitoring and maintaining vital signs, establishing a venous channel, cardiopulmonary resuscitation, respiratory tract management (including clearing the airway, trachea cannula placement, and the application of a respirator). The drug therapies included medications for antihypertension, decreasing intracranial pressure, and antigastrointestinal bleeding.

#### Construction of the HICH Knowledge Graph

We established the HICH knowledge graph (HKG) based on the Apache Jane triplet database and the RDF structured query language SPARQL. The HKG is able to extract, integrate, and align general medical information and HICH diagnostic and treatment-related data to enhance clarity of structure and improve retrieval performance. It serves as an external knowledge brain to augment text recognition and automated decision-making capabilities.

The HKG covers 3 dimensions of medical knowledge: general medical knowledge from medical textbooks; medical subdomain knowledge from clinical guidelines and expert consensus on common neurosurgical emergencies; and medical subdomain-specific knowledge from the hypertensive intracerebral hemorrhage weight system (HWS; detailed in Supplemental Methods 4 in Multimedia Appendix 3).

**Figure 2.**
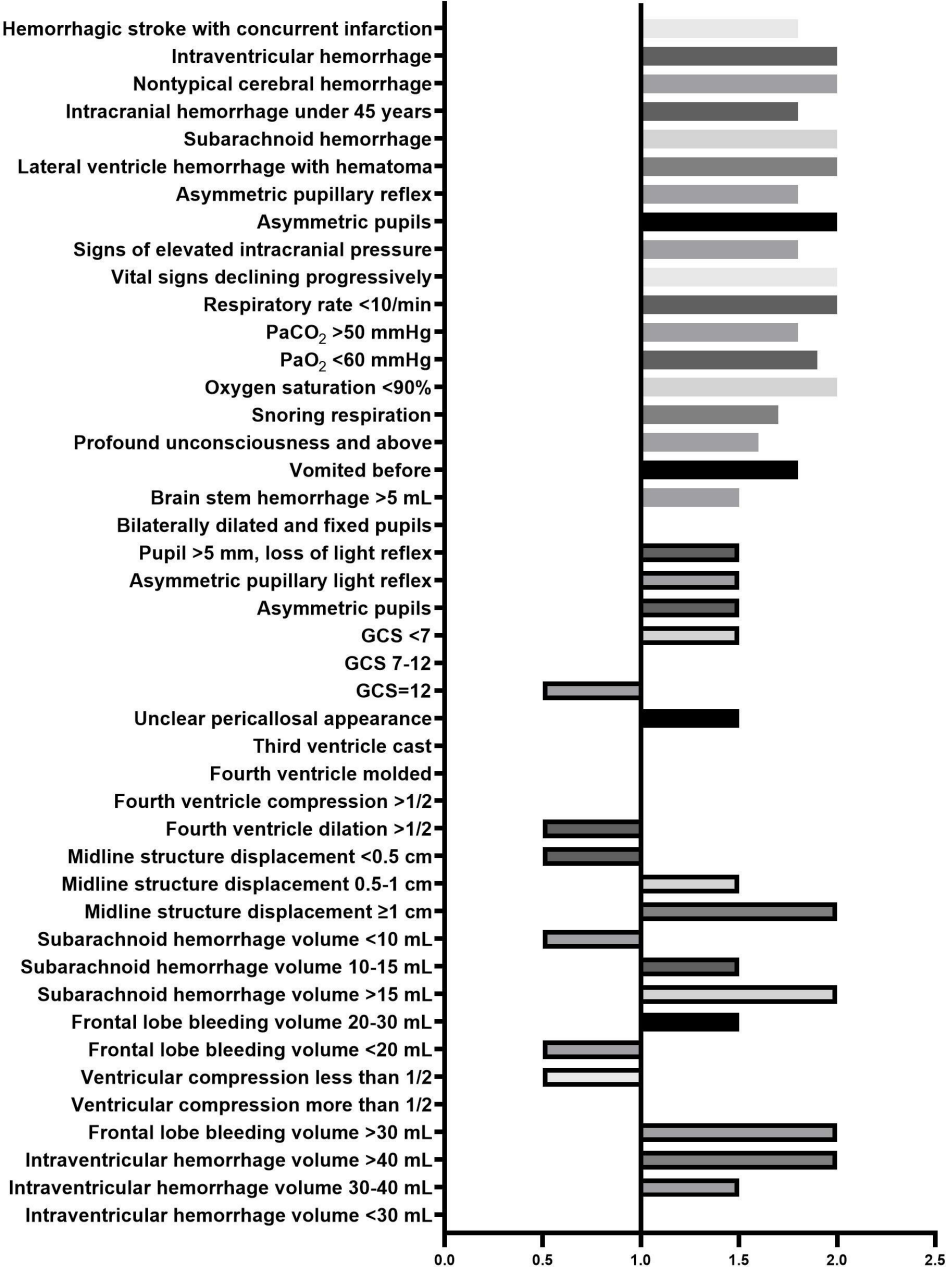
Some examples of HWS details. GCS: Glasgow Coma Score; HWS: hypertensive intracerebral hemorrhage weight system; Pa: partial pressure.

The HWS was designed based on the latest guidelines and clinical experience on HICH (Figure 2) [6,25-30]. The relationships among different variables were analyzed to establish the mapping relation between key information and treatment plans. Then, a total of 2000 HICH cases were randomly selected from the HICH-IT to train, validate, and test the HWS (detailed in Figure S2 in Multimedia Appendix 3).

#### Construction of the H-SYSTEM

**Figure 3.**
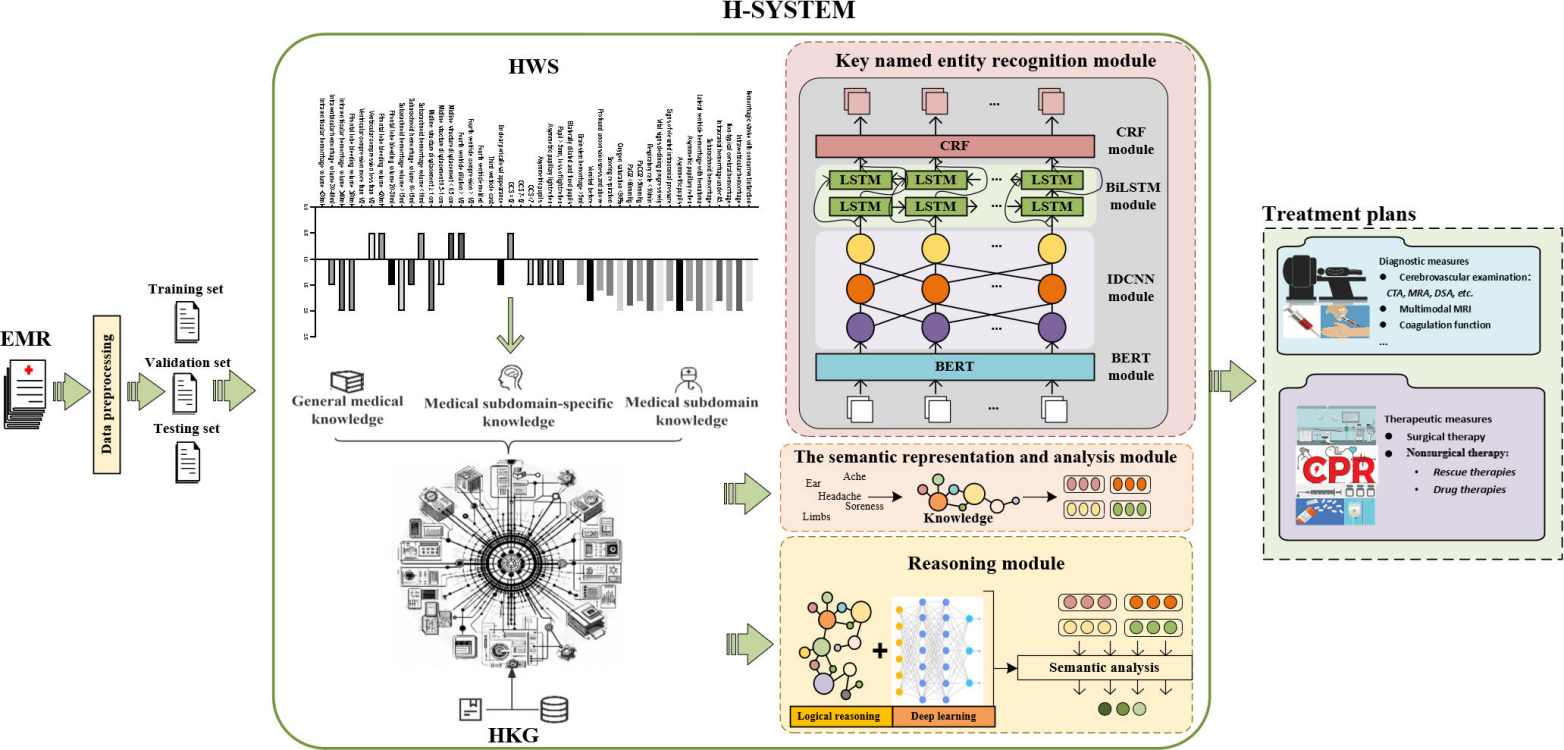
Construction and workflow of the H-SYSTEM. BERT: bidirectional encoder representations from transformers; BiLSTM: bidirectional long short-term memory; CRF: conditional random fields; CTA: computed tomography angiography; DSA: digital subtraction angiography; EMR: electronic medical system; HKG: hypertensive intracerebral hemorrhage knowledge graph; HWS: hypertensive intracerebral hemorrhage weight system; IDCNN: inflated dilated convolutional neural network; MRA: magnetic resonance angiography; MRI: magnetic resonance imaging.

The H-SYSTEM comprises 3 main modules (Figure 3): the key named entity recognition (NER) module, the HKG-enhanced semantic representation and analysis module, and HKG-enhanced reasoning module. These modules were meticulously designed and developed through collaborative work by a team of AI experts and neurosurgeons. A total of 3500 HICH cases were randomly sampled from the HICH-IT to establish the H-SYSTEM (training set=1500; validation set=1000; testing set=1000).

#### The Key NER Module

##### Overview

In this study, 7 key NER models were constructed based on 4 major modules: bidirectional encoder representations from transformers (BERT), inflated dilated convolutional neural network (IDCNN), bidirectional long short-term memory (BiLSTM), and conditional random fields (CRF; the descriptions of these models are detailed in Multimedia Appendix 3). The models were evaluated in the same experimental setting. Three metrics—precision (P), recall (R), and *F*_1_-score—were used to evaluate these models. After that, the NER model with best performance was chosen as the text recognition core of the H-SYSTEM.

##### BERT Model

The network architecture diagram of the BERT model is shown in Figure 3. The BERT achieves its pretraining objective through a masked language model and self-attention mechanism, extracting word embeddings for named entities as feature representations across the entire sequence. The calculation of this process is presented in Equation 1:


(1) $$Attention\;\left(Q,K,V\right)=softmax\left(K^{T}Q{\sqrt{d_{k}}}^{-1}\right)V$$

##### IDCNN Model

IDCNN uses dilated convolutions, which reduce data loss and expand the perceptual range by adjusting the dilation width of the convolution kernel, enabling the capture of longer text. The receptive field calculation formula for dilated convolutions is as follows:


(2) $$F_{i+1}={\left(2^{i+2}-1\right)}^{2}$$

##### BiLSTM Model

BiLSTM uses gating mechanisms such as input gates, output gates, and forgetting gates to achieve bidirectional capturing of global contextual features. The update equations for the states of the 3 gates are provided in Equations3^8^ below.


(3) $$f_{t}=\sigma_{g}(W_{f}*[h_{t-1},u_{t}]+b_{f})$$

(4) $$i_{t}=\sigma_{g}(W_{i}*[h_{t-1},u_{t}]+b_{i})$$

(5) $$O_{t}=\sigma_{g}(W_{o}*\left[h_{t-1},u_{t}\right]+b_{o})$$

(6) $${\tilde{C}}_{t}=\sigma_{h}(W_{c}*[h_{t-1},u_{t}]+b_{c})$$

(7) $$C_{t}=f_{t}{*C}_{t-1}+i_{t}*{\tilde{C}}_{t}$$

(8) $$h_{t}=O_{t}*\sigma_{g}\left(C_{t}\right)$$

##### CRF Model

Rather than treating each tag independently, the modeling of tag sequences is achieved through the utilization of CRF, enabling the joint modeling of tag sequences for a given input word sequence. This distribution is formulated as follows:


(9) $$P(Y|X)=\frac{1}{R(X)}\prod_{t=1}^{n+1}\;exp\left\{W_{t}\left(Y_{t-1},Y_{t}|X\right)\right\}$$

(10) $$R(X)=\sum_{Y\in Y(x)}\;\prod_{t=1}^{n+1}\;exp\left\{W_{t}\left(Y_{t-1},Y_{t}|X\right)\right\}$$

(11) $$W_{t}\left(Y_{t-1},Y_{t}|X\right)=\sum_{k=1}^{K}\;w_{k}f_{k}\left(Y_{t-1},Y_{t}|X\right)$$

In addition, the equations for calculating R, P, and *F*_1_-score are provided here:


(12) $$R=\frac{TP}{TP+FN}$$

(13) $$P=\frac{TP}{TP+FP}$$

(14) $$F_{1}=\frac{2PR}{P+R}$$

Where TP is true positive, FN is false negative, and FP is false positive.

### The Semantic Representation and Analysis Module

We used HKG to enhance the semantic representation module in analyzing medical key named entities retrieved from the EMRs of patients with HICH, the relationships between entities, and the potential semantic associations between entities and relationships.

To further analyze the features of medical key named entities, we established a Word2vec model to vectorize relevant medical texts. We established mappings between medical key named entities and measured the similarity of medical texts (Multimedia Appendix 3). The sentence-level representation and the cosine similarity between 2 vectors were obtained using the following formulas:


(15) $$V_{s}=\frac{\sum_{i=1}^{m}V_{i}*e^{w/(i)}}{m}$$

(16) $$cosim\left(V_{s1},V_{s2}\right)=\frac{V_{s1}*V_{s2}}{∥V_{s1}∥*∥V_{s2}∥}$$

### The Reasoning Module

This module combines logical reasoning with deep learning semantic similarity matching techniques, using an end-to-end pipeline model that integrates deep learning methods with prior knowledge. It uses natural language processing regularization techniques and deep learning semantic similarity matching techniques for logical diagnosis of entities. Furthermore, with the enhancement of HKG, the model gained stronger reasoning ability, and it output more explainable decisions.

### Assessment of the H-SYSTEM

The original treatment plan for the HICH cases was set as the gold standard, and 2 neurosurgeons (with more than 20 years of working experience) blinded to the processing conditions assessed the quality of the outputs of the H-SYSTEM using the following scoring standard. The whole treatment plan (full score=100 points) was divided into diagnostic measures (full score=15 points) and therapeutic measures (full score=85 points; detailed in Multimedia Appendix 3). In the event of a disagreement between the 2 neurosurgeons, the final verdict was made by a chief neurosurgeon with more than 25 years of working experience. Interrater reliability was assessed with κ statistics.

Then, the sensitivity, specificity, accuracy, positive predictive value, negative predictive value, and area under the curve (AUC) of the output were calculated and analyzed. Furthermore, the performance of the H-SYSTEM was also evaluated by analyzing the points distribution of treatment plans. The average time required for processing HICH cases by the H-SYSTEM was calculated and analyzed.

Meanwhile, we conducted a comparative analysis between the H-SYSTEM and ChatGPT (version 4.0) by inputting 300 randomly selected HICH cases into the 3 systems. Moreover, an additional 605 cases recently admitted to 6 medical centers from different regions were processed by the H-SYSTEM to test the practical application efficacy and generalization of the H-SYSTEM.

### Statistical Analyses

Data were analyzed with SPSS software (version 25.0 for Windows; IBM Corp) and GraphPad Prism (version 8.0 for Windows; GraphPad Software). Categorical variables are expressed as absolute numbers and percentages, and continuous variables are expressed as mean (SD). The sensitivity, specificity, positive predictive value, negative predictive value, and receiver operating characteristic curve were used to evaluate the performance of the H-SYSTEM. The AUC was used to measure the efficiency of different groups. Interrater agreement was measured using Cohen κ value. Accuracy was calculated to evaluate the performance of the H-SYSTEM. Two-tailed *P*<.05 was considered statistically significant. When calculating accuracy, sensitivity, specificity, and AUC, the following events were classified as positive events: reexamining head CT, surgical intervention, airway clearance, and intracranial pressure–lowering treatment.

## Results

### Reliability Analysis of HWS

#### Comparison With the Gold Standard

**Figure 4.**
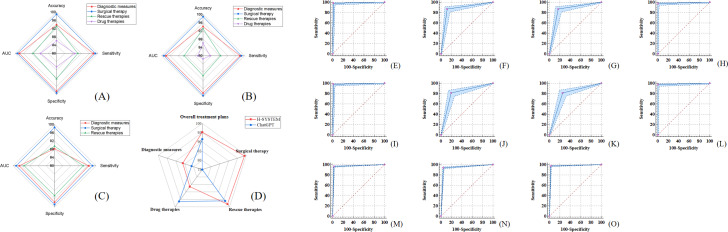
Comparison of HD, ND, ChatGPT, and the H-SYSTEM. (A-B) For both ND and HD, the diagnostic measures, drug therapies, surgical therapy, and rescue therapies had a high accuracy, sensitivity, specificity, and AUC. (C) In the testing set, the diagnostic measures, surgical therapy, and rescue therapies output by the H-SYSTEM had a high accuracy, sensitivity, specificity, and AUC. (D) In terms of accuracy, whether in overall assessment or in individual evaluations of diagnostic measures, rescue therapies, surgical therapy, and drug therapies, the H-SYSTEM consistently outperformed ChatGPT. (E-H) The ROC curves representing rescue therapies, surgical therapies, drug therapies, and diagnostic measures in the results output by the HD. (I-L) The ROC curves representing rescue therapies, surgical therapies, drug therapies, and diagnostic measures in the results output by the ND. (M-O) The ROC curves for the rescue therapies, surgical therapies, and diagnostic measures indicated the H-SYSTEM exhibited satisfactory and reliable performance in the testing set. AUC: area under the curve; HD: nonneurosurgical doctors who rely on the medical domain knowledge graph for hypertensive intracerebral hemorrhage; ND: neurosurgical doctor; ROC: receiver operating characteristic.

By setting the original treatment plans for the HICH cases as the gold standard, the overall accuracy of treatment plans made by nonneurosurgical doctors who rely on the HKG (HD) was 94.87%. Moreover, for diagnostic measures, rescue therapies, and surgical therapy, the accuracy, sensitivity, specificity, and AUC of HD were above 90%, while for drug therapies, these indexes of HD were above 85% (Table 1 and Figure 4). These results indicate the HWS is accurate and reliable.

**Table 1.** Comparison of the treatment plans of HD and ND when handling the same 800 cases. There were no statistically significant differences observed between the two in terms of diagnostic measures, surgical therapy, rescue therapies, or drug therapies.^a^^,^^b^

	HD					ND				
	Overall treatment plans	Diagnostic measures	Surgical therapy	Rescue therapies	Drug therapies	Overall treatment plans	Diagnostic measures	Surgical therapy	Rescue therapies	Drug therapies
Mean accuracy (95% CI), %	94.87 (94.58‐95.15)	93.89 (94.59‐95.14)	98.94 (98.08‐99.81)	93.14 (91.99‐94.28)	86.10 (76.05‐96.14)	94.86 (94.59‐95.14)	94.13 (90.63‐97.63)	98.89 (98.41‐99.37)	93.09 (92.01‐94.18)	86.06 (75.50‐96.62)
Mean sensitivity (95% CI), %	N/A^c^	97.77 (95.32‐100)	98.70 (96.12‐100)	90.27 (80.87‐99.66)	88.80 (84.41‐93.19)	N/A	97.80 (94.79‐100)	98.50 (96.35‐100)	88.43 (72.57‐100)	83.33 (78.83‐87.84)
Mean specificity (95% CI), %	N/A	98.17 (96.05‐100)	99.10 (97.31‐100)	92.27 (83.50‐100)	86.50 (77.55‐9545)	N/A	98.03 (97.09‐98.97)	99.23 (98.23‐100)	89.60 (69.88‐100)	81.60 (66.06‐97.14)
Mean area under the curve (95% CI), %	N/A	97.93 (96.44‐99.43)	98.90 (98.00‐99.80)	91.27 (82.75‐99.78)	87.70 (81.67‐93.73)	N/A	97.90 (95.93‐99.87)	98.87 (98.29‐99.44)	89.10 (70.98‐100)	82.47 (72.36‐92.57)

a HD: nonneurosurgical doctors who rely on the HWS (the hypertensive intracerebral hemorrhage weight scoring system) for their output results.

b ND: senior neurosurgical doctors.

c N/A: not applicable.

#### Comparison With ND

The results showed that the accuracy of overall treatment plans made by ND was lower than that of HD. For diagnostic measures, the accuracy of ND was slightly higher than that of HD, while for the surgical therapies and rescue therapies, the accuracy of HD was slightly higher than that of ND. However, the above differences were not statistically significant (94.86% vs 94.87%, 94.13% vs 93.89%, 98.94% vs 98.89%, 93.14% vs 93.09%; *P*>.05; Table 1).

#### Comparison of the Performance of HD and ND

As shown in Table 2, the concordance between the points distributions of overall treatment plans (100%) output by HD and ND was satisfactory (κ=0.730). Moreover, for diagnostic measures, surgical therapies, rescue therapies, and drug therapies, the concordance between the points distributions of HD and ND was also satisfactory (κ=0.805, 0.715, 0.890, 0.799; Table 2 and Figure 4). Meanwhile, the H-SYSTEM also output the basis of treatment plans (detailed in Multimedia Appendix 3).

**Table 2.** The reliability analysis of the hypertensive intracerebral hemorrhage weight scoring system. The results generated by ND and HD for the same 800 cases were scored, and an analysis and comparison were conducted based on the distribution of scores.^a^^,^^b^

	HD, mean (SD), %	ND, mean (SD), %	*P* value	κ
**Overall treatment plans (100 points)**				0.730
>80 points	97.61 (0.29)	97.72 (0.31)	.81	
60‐80 points	1.50 (0.17)	1.39 (0.29)	.76	
<60 points	0.89 (0.20)	0.89 (0.15)	>.99	
**Diagnostic measures (15 points)**				0.805
>80% (12 points)	85.94 (2.20)	78.56 (5.42)	.28	
60%‐80% (9‐12 points)	11.89 (2.02)	18.72 (5.64)	.32	
<60% (9 points)	2.17 (0.19)	2.72 (0.40)	.28	
**Therapeutic measures (85 points)**				
* * **Surgical therapy (40 points)**				0.715
100% (40 points)	98.89 (0.11)	98.94 (0.20)	.82	
0% (0 point)	1.11 (0.11)	1.06 (0.20)	.82	
**Rescue therapies (35 points)**				0.890
>80% (28 points)	92.67 (0.17)	92.50 (0.17)	.5	
60%‐80% (21‐28 points)	4.28 (1.07)	4.28 (1.07)	>.99	
<60% (21 points)	2.11 (0.06)	2.28 (0.20)	.47	
**Drug therapies (10 points)**				0.799
>80% (8 points)	72.44 (7.96)	72.00 (8.01)	.97	
60%‐80% (6‐8 points)	23.61 (5.55)	23.78 (5.84)	.98	
<60% (6 points)	3.94 (2.44)	4.22 (2.34)	.94	

a ND: senior neurosurgical doctors.

b HD: nonneurosurgical doctors who rely on the HWS (the hypertensive intracerebral hemorrhage weight scoring system) for their output results.

### Reliability Analysis of the Key NER Model

**Figure 5.**
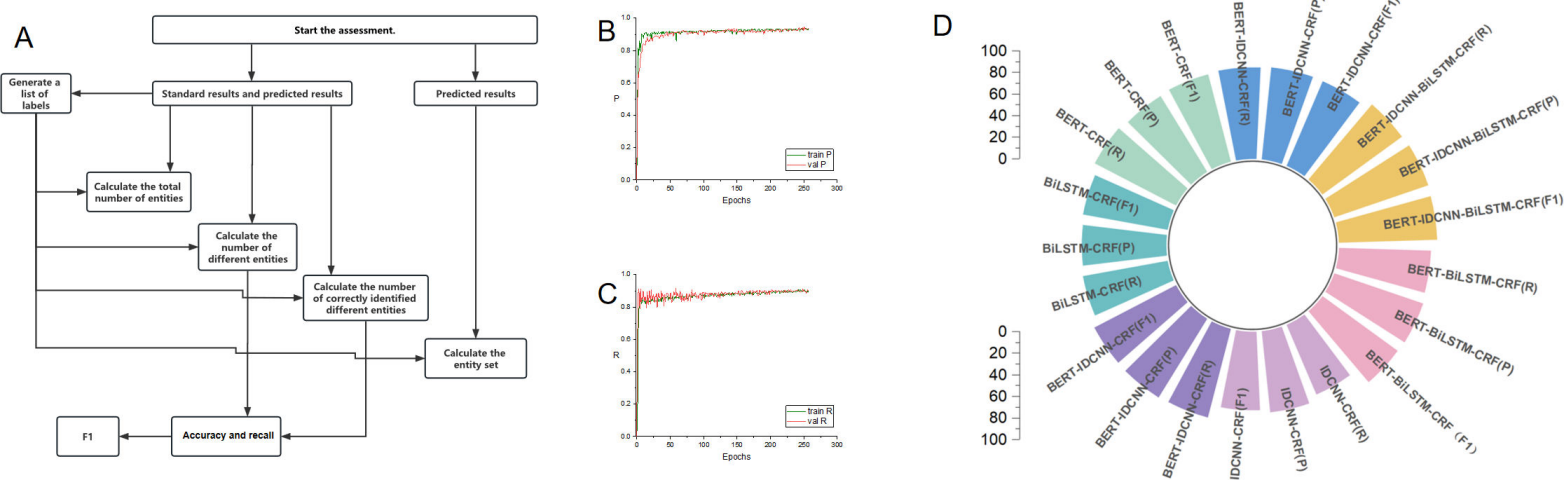
The training and evaluation process of the key named entity recognition module. (A) The validation process of the key named entity recognition module. (B-C) The change curves of P and R with the increase in iteration times. As the iterations progress, both P and R gradually reach a stable state. (D) Comparison between the BERT-IDCNN-BiLSTM-CRF model and other models in terms of P, R, and *F*_1_-score. The BERT-IDCNN-BiLSTM-CRF model outperforms the other models in terms of precision, recall, and *F*_1_-score, indicating it not only achieves better precision and recall in key named entity recognition, but also maintains a balanced performance between the 2 metrics. The P measures the proportion of actual positive samples among all samples predicted as positive by the model; R measures the proportion of successfully predicted positive samples among all actual positive samples. The *F*_1_-score, being the harmonic mean of P and R, indicates stronger model performance as its value approaches 1. BERT: bidirectional encoder representations from transformers; BiLSTM: bidirectional long short-term memory; CRF: conditional random fields; IDCNN: inflated dilated convolutional neural network; P: precision; R: recall.

A total of 7 key NER models were constructed and evaluated, including IDCNN-CRF, BiLSTM-CRF, BiLSTM-IDCNN-CRF, BERT-CRF, BERT-IDCNN-CRF, BERT-BiLSTM-CRF, and BERT-IDCNN-BiLSTM-CRF. The P, R, and *F*_1_ values for the BERT-IDCNN-BiLSTM-CRF model were reported as 92.03, 90.22, and 91.11, which were significantly higher than those of the other models. These findings indicated that the BERT-IDCNN-BiLSTM-CRF model not only achieved better precision and recall in key NER but also maintained a balanced performance between the 2 metrics (Figure 5 and Table 3).

**Table 3.** The comparative analysis of data across various models. All metrics of our approach are higher than those of other models.

Models	Precision	Recall	*F*_1_-score
IDCNN^a^-CRF^b^	75.54	70.32	72.83
BiLSTM^c^-CRF	79.80	77.16	78.45
BiLSTM-IDCNN-CRF	78.16	72.37	75.15
BERT^d^-CRF	84.81	82.45	83.61
BERT-IDCNN-CRF	85.68	84.84	85.25
BERT-BiLSTM-CRF	87.04	85.31	86.16
BERT-IDCNN-BiLSTM-CRF (our approach)	92.03	90.22	91.11

a IDCNN: inflated dilated convolutional neural network.

b CRF: conditional random fields.

c BiLSTM: bidirectional long short-term memory.

d BERT: bidirectional encoder representations from transformers.

The iteration curve of the BERT-IDCNN-BiLSTM-CRF model is depicted in Figures 5B and 5C. After approximately 200 iterations, there was no noteworthy enhancement observed in both P and R values for both the training and validation sets. This suggests that the model has attained its optimum and stable state. The BERT pretrained model exhibited robust semantic representation capabilities, which contributed to its excellent performance in downstream recognition tasks and significantly enhanced the effectiveness of the model in NER.

### Reliability Analysis of the H-SYSTEM

#### Overall Test of the H-SYSTEM

The accuracy of treatment plans output by the H-SYSTEM was 91.74%. which also showed significant consistency with the gold standard (*P*<.05). For diagnostic measures, the accuracy, AUC, and κ were 88.18%, 97.03%, and 0.874, respectively (*P*<.05). For surgical therapy, these indexes were 98.53%, 98.53%, and 0.971 (*P*<.05). For rescue therapies, these indexes were 89.50%, 94.67%, and 0.923, respectively (*P*<.05; Tables4  5, Figure 4). The detailed treatment plans of the gold standard and the H-SYSTEM are displayed in Multimedia Appendix 3*.*

**Table 4.** Comparison of the performance of doctors using the H-SYSTEM and ND.^a^^,^^b^

	^c^H-SYSTEM				ND			
	Overall treatment plans	Diagnostic measures	Surgical therapy	Rescue therapies	Overall treatment plans	Diagnostic measures	Surgical therapy	Rescue therapies
Mean accuracy (95% CI), %	94.87 (94.58‐95.15)	93.89 (94.59‐95.14)	98.94 (98.08‐99.81)	93.14 (91.99‐94.28)	94.86 (94.59‐95.14)	94.13 (90.63‐97.63)	98.89 (98.41‐99.37)	93.09 (92.01‐94.18)
Mean sensitivity (95% CI), %	N/A^d^	97.77 (95.32‐100)	98.70 (96.12‐100)	90.27 (80.87‐99.66)	N/A	97.80 (94.79‐100)	98.50 (96.35‐100)	88.43 (72.57‐100)
Mean specificity (95% CI), %	N/A	98.17 (96.05‐100)	99.10 (97.31‐100)	92.27 (83.50‐100)	N/A	98.03 (97.09‐98.97)	99.23 (98.23‐100)	89.60 (69.88‐100)
Mean area under the curve (95% CI), %	N/A	97.93 (96.44‐99.43)	98.90 (98.00‐99.80)	91.27 (82.75‐99.78)	N/A	97.90 (95.93‐99.87)	98.87 (98.29‐99.44)	89.10 (70.98‐100)

a To enhance the validation of the performance of the H-SYSTEM, we input 1000 cases into the H-SYSTEM, compared its output with that of ND, and assessed their accuracy, sensitivity, specificity, and area under the curve through calculations and comparisons.

b ND: senior neurosurgical doctors.

c HD: nonneurosurgical doctors who rely on the HWS (the hypertensive intracerebral hemorrhage weight scoring system) for their output results.

d N/A: not applicable.

**Table 5.** The reliability analysis of the H-SYSTEM and the distribution of scores on different treatment plans output by the H-SYSTEM and ND.^a^

	H-SYSTEM, mean (SD), %	ND, mean (SD), %	*P* value	κ
**Overall treatment plans (100 points)**				0.841
>80 points	94.90 (0.404)	92.77 (0.73)	.06	
60‐80 points	3.73 (0.52)	4.60 (0.30)	.06	
<60 points	1.37 (0.15)	2.63 (0.53)	.20	
**Diagnostic measures (15 points)**				0.874
>80% (12 points)	77.57 (0.09)	75.17 (0.90)	.11	
60%‐80% (9‐12 points)	17.53 (0.27)	18.13 (0.93)	.64	
<60% (9 points)	4.90 (0.26)	6.70 (0.10)	.02	
**Therapeutic measures (85 points)**				
***Surgical therapy (40 points)***				0.221
100% (40 points)	98.60 (0.12)	98.53 (0.15)	.80	
0% (0 points)	1.40 (0.12)	1.47 (0.15)	.80	
***Rescue therapies (35 points)***				0.923
>80% (28 points)	88.00 (0.36)	87.13 (0.46)	.32	
60%‐80% (21‐28 points)	8.83 (0.38)	9.17 (0.43)	.63	
<60% (21 points)	3.17 (0.07)	3.70 (0.06)	.03	

a ND: senior neurosurgical doctors.

**Figure 6.**
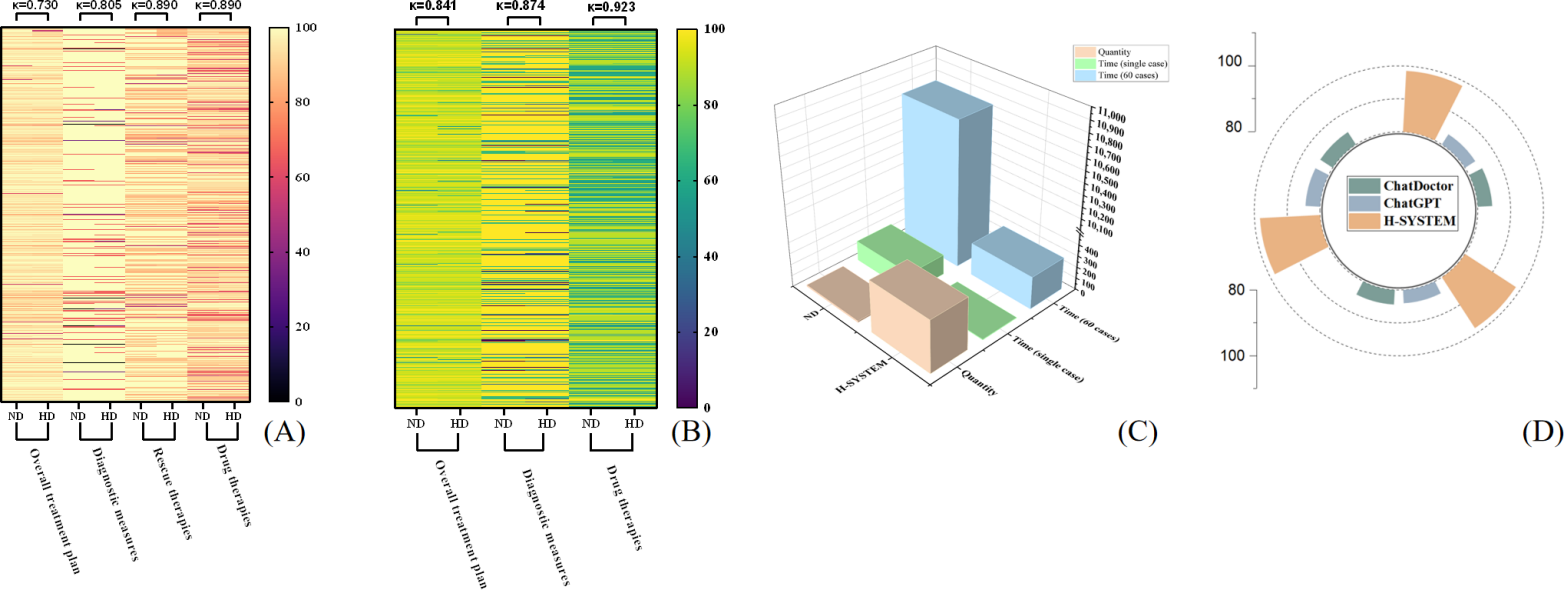
Comparison of output results of HD, ND, H-SYSTEM, ChatGPT, and ChatDoctor. (A) The score distributions of treatment plans output by ND and HD showed no significant difference. For overall treatment plans, diagnostic measures, surgical therapies, rescue therapies, and drug therapies, the concordance between the points distributions of HD was satisfactory. (B) The score distributions of treatment plans output by the H-SYSTEM and ND showed no significant difference. The H-SYSTEM has an accuracy rate of over 80% in the majority of cases, and there is a high level of consistency between the results produced by the H-SYSTEM and ND. (C) Comparison of comprehensive efficiency between the H-SYSTEM and ND. (D) Quantitative comparison of ChatDoctor, ChatGPT, and the H-SYSTEM with BERTScore. HD: nonneurosurgical doctors who rely on the medical domain knowledge graph for hypertensive intracerebral hemorrhage; ND: neurosurgical doctor.

The score distributions of treatment plans output by the H-SYSTEM and ND showed no significant difference. The concordance between the 2 raters on the independent testing dataset of the H-SYSTEM and ND output was satisfactory (κ=0.841). In the evaluation of the qualification rate for the distribution of the overall points, there was no statistically significant difference between the H-SYSTEM and ND output. Furthermore, the majority of both HD and ND output results had an accuracy rate exceeding 80%. The difference between ND and HD remained statistically insignificant (Tables2  5, Figure 6).

#### Comprehensive Effectiveness

For ND, the total time spent on 60 cases and a single case was significantly longer as compared with the H-SYSTEM. Obviously, the speed of the H-SYSTEM in processing HICH cases was significantly higher than that of ND (*P*<.001). However, no statistical difference in the sensitivity, specificity, accuracy, and AUC was found between the treatment plans of doctors and the H-SYSTEM (*P*>.05; Figure 6, Tables S2 and S3 in Multimedia Appendix 3).

#### Practical Application Efficacy and Generalization of the H-SYSTEM

The results showed that ChatGPT achieved an overall accuracy of 91.48% (95% CI 90.14%‐92.77%), which is significantly lower than that of the H-SYSTEM (95.26%, 95% CI 94.43%‐96.83%). Meanwhile, for diagnostic measures, surgical therapies, rescue therapies, and drug therapies, the H-SYSTEM also had significantly higher accuracy as compared with ChatGPT (Table 6). Therefore, for patient medical text processing and decision-making, the H-SYSTEM surpassed ChatGPT with statistical significance (*P*<.05).

**Table 6.** Comparison between the H-SYSTEM and ChatGPT (N=300 cases). The accuracy of the H-SYSTEM was significantly higher than that of ChatGPT.

	ChatGPT, mean (95% CI), %	H-SYSTEM, mean (95% CI), %	*P* value
Overall treatment plans	91.48 (90.14‐92.77）	95.26 (94.43‐96.83）	<.01
Diagnostic measures	75.00 (70.07‐79.93)	86.11 (82.19‐90.03)	<.001
Surgical therapies	96.00 (93.77‐98.23）	99.00 (97.87‐100）	.02
Rescue therapies	96.36 (95.40‐97.31）	98.16 (97.64‐98.67）	.002
Drug therapies	81.08 (77.84‐84.33）	86.44 (84.72‐88.15）	.006

In another study, a medical chat model fine-tuned on a large language model meta-AI (LLaMA) named ChatDoctor showed higher P, R, and *F*_1_-scores (84.44, 84.51, and 84.46, respectively) as compared with ChatGPT (83.7, 84.45, and 84.06, respectively) when processing medical text tasks [31]. Similarly, in our study, the P, R, and *F*_1_-score of the H-SYSTEM were 98.53, 98.33, and 98.43, which were all higher than that of ChatGPT (96.00, 77.00, and 85.46, respectively).

An additional 605 patients recently admitted to 6 different medical centers from different regions were included to further test the practical application efficacy of the H-SYSTEM. The results showed that the total accuracy of treatment plans output by the H-SYSTEM was 92.22% (range: 91.14%-95.35%; Table 7).

**Table 7.** Performance of the H-SYSTEM on HICH electronic medical records from different medical centers. The accuracies of the H-SYSTEM on HICH cases from different medical centers ranged from 91.14% to 95.35%.^a^

	Quantity of HICH cases	Overall treatment plans, mean (95% CI), %
Overall medical centers	605	92.22 (91.57‐92.88)
Medical center 1 (Chongqing)	216	92.04 (90.95‐93.13)
Medical center 2 (Chongqing)	128	91.93 (90.41‐93.35)
Medical center 3 (Sichuan)	95	92.18 (90.22‐94.15)
Medical center 4 (Yunnan)	73	91.14 (89.10‐93.18)
Medical center 5 (Yunnan)	62	93.23 (91.90‐94.57)
Medical center 6 (Shanxi)	31	95.35 (93.88‐96.83)

a HICH: hypertensive intracerebral hemorrhage.

## Discussion

### Principal Findings

Despite increasing attention being directed to the research and application of AI in medicine [23,32], several challenges remain substantial obstacles to the development and translation of medical AI systems into clinical practice. The first challenge is access to large and well-annotated datasets. The second challenge is that the inner workings and decision-making processes of machine-learning algorithms remain opaque, which is also called the black box effect. In addition, although AI models like LLMs have shown excellent performance in various tasks, they do not perform as well as expected in clinical medical practice due to the highly abstract and specialized medical domain knowledge required and the complexity of clinical tasks.

To address these challenges, we developed a medical domain knowledge graph–enhanced automatic decision support system, named H-SYSTEM, based on adequate data from 15 medical centers. Meanwhile, the experienced neurosurgeon-AI team ensured that the H-SYSTEM obtained large and well-annotated datasets and had a high generalization capacity. The H-SYSTEM identifies and analyzes data from patients’ medical history, physical examination, and auxiliary examination, then outputs the treatment plans; it also provides the basis for these decisions (Figure 3). Different from LLMs and routine deep learning systems [23,32-34], the H-SYSTEM is enhanced by a medical domain knowledge graph (called HKG), which means that it can efficiently output both explainable and elaborate clinical decisions (Multimedia Appendix 2). One highlight of the H-SYSTEM is its ability to explain the rationale of its decisions, helping the neurosurgeons understand its inner working process. Thus, this understandable system can gain more trust from doctors and facilitate both the development and adoption of AI systems in clinical practice.

The HKG in this study covers medical knowledge from 3 dimensions: the general medical domain knowledge, the medical subdomain knowledge, and the medical subdomain-specific knowledge. The treatment plans based on the HKG showed high reliability as compared with doctors. Therefore, the HKG was a reliable guide and external knowledge brain for the H-SYSTEM. It enhanced not only the capability of the semantic analysis and representation model to handle clinical tasks but also the explainability of the decision output by the H-SYSTEM.

The key NER model, BERT-IDCNN-BiLSTM-CRF, exhibited excellent performance with fast convergence and efficient extraction of key named entities, and it outperformed other NER models in this study (Table 3 and Figure 4). Deep learning–based models offer ample representability for text, enabling efficient problem-solving with high-quality outcomes. Meanwhile, the word vector space enhanced by the HKG proved to be more suitable for medical scenarios (Multimedia Appendix 3). These models efficiently encode information from the data, facilitating the effective utilization of available data in the model.

The H-SYSTEM in this study is designed to imitate the process and thinking style of a neurosurgeon during HICH diagnosis and treatment in the emergency room. Like a neurosurgeon, the system analyzes all the medical records that can be obtained in the emergency room, including chief complaints, current medical history, important past illness history, physical examination, and important auxiliary examinations. Compared with the AI system in our previous study [34], the H-SYSTEM in this study has a more powerful ability in key NER, semantic analysis, and logical decision-making. This enables it to process more complicated HICH cases and output more elaborate treatment plans that are practical in the emergency room. Besides outputting elaborate treatment plans, the H-SYSTEM also displays the basis of making these treatment plans. Our approach mimics the workflow of neurosurgeons in clinical practice and enables the users to independently review the basis of the H-SYSTEM’s recommendations. This provides the rationale or support for its decisions and helps the human users understand the inner working process of the H-SYSTEM. Therefore, the reliability of the H-SYSTEM is significantly increased, which may help it gain the trust of neurosurgeons.

The treatment plans output by the H-SYSTEM consist of diagnostic and therapeutic measures. For patients with a complicated past medical history or unclear diagnosis, diagnostic measures are crucial for the whole treatment process. For instance, if a patient with intracerebral hemorrhage is suspected of having cerebrovascular diseases or a tumor-associated hemorrhage, then cerebrovascular-related examinations like computed tomography angiography, magnetic resonance angiography, or digital subtraction angiography, or multiple-modal magnetic resonance imaging, are included in their diagnostic measures to verify or exclude cerebrovascular diseases and brain tumors. Furthermore, for patients with a complicated past medical history, such as hemophilia, anticoagulant therapy, or hemodialysis, blood coagulation function examinations are included in their diagnostic measures to evaluate the bleeding and rebleeding risk in subsequent treatments.

Compared with the diagnostic measures, the therapeutic measures designed in the study were more complicated and elaborate. For instance, the respiratory tract management in the rescue therapy consisted of several measures, including cleaning up the airway, tracheal cannula insertion, and application of a respirator. Each measure had elaborate scoring rules and thresholds that determined whether the measure would be implemented or not. Two situations are the most concerning for a neurosurgeon in the emergency room—the vital signs and surgical indications of patients with HICH—and the performance of the H-SYSTEM was reliable for both rescue therapies and surgical therapies. Meanwhile, the efficiency of the H-SYSTEM in processing HICH cases was significantly higher than that of a neurosurgeon, indicating that the H-SYSTEM can provide reliable and efficient support in the diagnosis and treatment of HICH (Figure 6, Table S1 in Multimedia Appendix 3).

During the comparison with 2 LLMs, the H-SYSTEM also showed excellent performance. Although ChatGPT performed well in some relatively simple HICH cases, its output treatment plans are more like general management principles. Moreover, when facing complicated HICH cases that had a changeful condition, complicated medical history, or atypical clinical and CT manifestation, the outputs of ChatGPT were not so accurate and did not offer substantial assistance and support to doctors. Meanwhile, the ChatDoctor, which has been fine-tuned on LLaMA, surpassed ChatGPT in multiple parameters by using medical domain knowledge [31]. Because ChatGPT is not open source, we cannot compare it with the H-SYSTEM directly. Therefore, we took ChatGPT as the medium and calculated the P, R, and *F*_1_-score. The results showed that the above values of ChatDoctor were higher than that of ChatGPT, but still lower than that of the H-SYSTEM. These results indicated that, without sufficient medical subdomain and subdomain-specific knowledge, even LLMs could not fully demonstrate their advantages in clinical text processing tasks.

In contrast, the H-SYSTEM is an HICH-specialized decision support system with dedicated diagnostic and treatment logics for HICH and a large training and validating database from multiple medical centers. Hence, it accurately processed complicated HICH cases and provided valuable assistance and support for doctors.

Furthermore, the HICH data in this study originated from 15 medical centers, covering an area of nearly 6 square kilometers in major provinces of western China, with a population of approximately 300 million. The EMRs of these HICH cases not only encompass different EMR systems of various medical centers but also reflect the diverse writing habits and academic and educational backgrounds of different doctors. This indicates that, in addition to its dependable clinical support for neurosurgeons, the H-SYSTEM also exhibits outstanding generalization capability.

Although the results of this study are promising, we have to acknowledge it does have some limitations. For the H-SYSTEM, the biggest challenge was to process and analyze the ambiguous EMRs. Due to various reasons, such as limitations of the EMR system, writing habits, and academic and educational backgrounds, some EMRs seem ambiguous, making it difficult to uncover the patient’s real information. Therefore, we plan to continuously expand the current dataset to cover more medical centers and regions. Moreover, more complex cases, which are also challenging for neurosurgeons, are still needed for training the system. Additionally, the H-SYSTEM still has room for improvement in providing more personalized diagnosis and treatment plans. Although the H-SYSTEM identified CT reports in the EMR with high accuracy, it could not directly recognize the CT image. Thus, some possible biases caused by different radiologists might be inevitable. In future studies, these possible biases will be addressed by integrating automatic image segmentation into the H-SYSTEM.

### Conclusions

This study developed H-SYSTEM, an AI-based decision support system for HICH, which outperformed existing models like ChatGPT and ChatDoctor, and showed strong generalization capabilities for potential clinical application.

The H-SYSTEM is based on multiple-center data and shows significantly high efficiency and generalization capacity in processing EMRs; it also provides explainable and elaborate treatment plans. Therefore, it has the potential to gain more trust from doctors and facilitate both the development and adoption of AI systems in clinical practice.

## Supplementary material

10.2196/66055 Multimedia Appendix 1 The annotated keywords in the electronic medical records of different hypertensive intracerebral hemorrhage cases.

10.2196/66055 Multimedia Appendix 2 Treatment plans: gold standard, ChatGPT, and the H-SYSTEM.

10.2196/66055 Multimedia Appendix 3 Supplemental methods and results.

## Abbreviations

AI: artificial intelligence
BERT: bidirectional encoder representations from transformers
BiLSTM: bidirectional long short-term memory
CRF: conditional random fields
CT: computed tomography
EMR: electronic medical record
HD: nonneurosurgical doctors who rely on the HKG
HICH: hypertensive intracerebral hemorrhage
HICH-IT: hypertensive intracerebral hemorrhage image and text database
HKG: HICH knowledge graph
HWS: hemorrhage weight system
ICH: intracerebral hemorrhage
IDCNN: inflated dilated convolutional neural network
LLaMA: large language model meta-AI
ND: neurosurgical doctor
NER: named entity recognition
