# Supplementary material for: Knowledge Graph–Enhanced Deep Learning Model (H-SYSTEM) for Hypertensive Intracerebral Hemorrhage: Model Development and Validation
Source: J Med Internet Res. 2025 Jun 12;27:e66055. doi: 10.2196/66055 (PMC12203281; doi:10.2196/66055)
Supplement: Multimedia Appendix 1 [file jmir-v27-e66055-s001.docx]

**Appendix 2 The annotated key words in the EMRs of different HICH cases**

**The words highlighted in green were the key words which were annotated by the neurosurgeon and AI researchers together.**

**Simple case：**

76-year-old female, chief complaint: “sudden right-sided weakness for over 4 hours”;

Present illness history: Four plus hours before admission, the patient experienced sudden right-sided weakness while using the restroom. Her right knee touched the ground, she was able to call for help, and reported no chest tightness, palpitations, dizziness, or shortness of breath. Upon being discovered by her family, she displayed reduced strength in her right-sided limbs and slightly slurred speech. After self-administering oxygen for one hour, her family noticed a worsening of the right-sided weakness and speech impairment. Throughout the course of the illness, she did not experience nausea, projectile vomiting, or other discomfort. She was brought to our emergency department three hours prior, and was admitted to the Intensive Care Unit for further treatment. Since the onset of this episode, the patient has maintained a regular appetite, clear consciousness, reasonable mental alertness, satisfactory sleep, normal bowel movements, normal urination, and no significant changes in weight.

Significant past medical history: None.

Personal history: Denies a history of living in an epidemic area or contact with contaminated water. Denies any history of drug allergies.Denies a history of smoking and alcohol consumption.

Family history: Parents are deceased. Denies a family history of hereditary diseases. Denies a family history of tumors.

Physical examination:

Temperature: 36.5°C, Pulse: 78 beats per minute, Respiratory Rate: 18 breaths per minute, Blood Pressure: 179/95 mmHg; Consciousness level: drowsiness, GCS score: 14, Left pupil: 3mm, sensitive to light reflex, Right pupil: 3mm, sensitive to light reflex, Muscle strength in right upper limb: Grade 2, Muscle strength in right lower limb: Grade 3, Normal movement in left limbs, no elicited pathological signs.

CT Report:

Head CT scan indicates a left basal ganglia area hemorrhage, estimated at approximately 14 ml, with no significant midline shift observed. Left lateral ventricle compression is less than one-third. Normal chest CT scan, no obvious abnormalities observed.

ECG Report:

No obvious abnormalities observed.

**Complex case：**

A 65-year-old female patient is presenting with a sudden headache accompanied by left-sided limb impairment that has persisted for over 5 hours.

Present illness history: Approximately 5+ hours prior to admission, the patient experienced an abrupt headache without any apparent cause. Alongside this, she developed left-sided limb impairment, while her right-sided limbs retained their functionality. She experienced vomiting once, with the vomit containing stomach contents. The patient is capable of providing basic responses and did not exhibit urinary or bowel incontinence or limb convulsions. Her family promptly dialed 120 (emergency services), and she was swiftly transported to our emergency department.

Significant past medical history:The patient has a 6-year history of hypertension, with the highest recorded blood pressure reaching 190+/100+ mmHg. Her medication adherence has been inconsistent, and there are no specific details regarding blood pressure management. There are no other significant medical history points.

Personal history: Denies a history of living in an epidemic area or contact with contaminated water. No history of drug allergies. Smoked for 15 years, averaging 5 cigarettes per day. Quit smoking for the past 5 years. Occasionally consumes a small amount of alcohol.

Family history: There is no family history of hereditary diseases, congenital disorders, or other infectious diseases.

Physical Examination:

Pulse: 65 beats per minute, Blood Pressure: 190/100 mmHg, Respiratory rate: 17 breaths per minute, Oxygen saturation: 96%, Level of consciousness: light coma, with a GCS score of 10, Pupillary responses: The left pupil measures 3mm and responds to light; the right pupil measures 3.5mm, with a sluggish light reflex, Left-sided limbs exhibit limited response to painful stimuli, whereas right-sided limbs demonstrate voluntary movement, Babinski reflex: Present on the left side and absent on the right side.

CT Report:

A head CT scan revealed a patchy high-density shadow in the right temporal lobe with a pronounced mass effect, indicating a potential hemorrhage with an estimated volume of about 40ml. High-density shadows were also identified in the right lateral fissure, ambient cistern, suprasellar cistern, and the adjacent subarachnoid space, suggestive of subarachnoid hemorrhage. There is an approximate 1.5cm leftward midline shift, significant compression of the right lateral ventricle, and the compression is about one-third. Chest CT shows a few inflammatory nodules, follow-up recommended.

ECG Report:

Sinus rhythm.

**Difficult and complicated case：**

54-year-old female, presenting with “sudden headache, vomiting, and altered consciousness for one day.”

Present illness history: One day before admission, the patient engaged in a heated argument with someone, after which she experienced sudden onset of headache and vomiting (with vomitus containing gastric contents). She subsequently collapsed to the ground, with the exact location of the fall unknown. After falling, she was able to move her left-sided limbs, but was unable to speak. She also exhibited weakness in her right-sided limbs, without any further vomiting, frothing at the mouth, or bleeding from the mouth, nose, or external ear canal. There were no limb convulsions or urinary or bowel incontinence. As a result, she sought medical attention at our hospital's emergency department and was admitted to our department with a diagnosis of “brain hemorrhage.”

Significant past medical history:The patient has a six-year history of hypertension, with the highest blood pressure recorded at 150/? mmHg. She has been taking unspecified oral medications intermittently for blood pressure management, with poor control. She has not taken antihypertensive medications recently. Four years ago, she was diagnosed with unstable angina and has been on long-term oral aspirin therapy at a dose of 100mg once daily.

Personal history: The patient was born in her hometown and has been a long-term resident in the local area. She deny any history of exposure to dust or toxins, as well as any history of sexually transmitted diseases or engaging in risky behavior. She got married at the age of 24 and have one daughter and two sons. Her spouse and children are in good health.

Family history: There is no known history of similar illnesses in her family, and there is no history of infectious or hereditary diseases. Her mother passed away from lung cancer four years ago.

Physical Examination: Temperature: 38.1℃. Pulse: 60 beats per minute. Respiratory Rate: 12 breaths per minute. Blood Pressure: 170/61 mmHg. Snoring respirations. Consciousness: Grade IV, GCS score of 8 points. Right pupil diameter: Approximately 3mm, reactive to light. Left pupil diameter: 4mm, sluggish response to light stimulation. Stimulation of the left lower limb produces visible movement, while the right limb shows no signs of movement. Bilateral Babinski sign is suspiciously positive.

CT Report:

Head CT reveals high-density shadows in the left basal ganglia region, suggestive of hematoma, with an estimated volume of approximately 39ml. Midline structures are shifted towards the opposite side by about 6mm. The left ventricle is compressed by more than half, and there are high-density shadows in the subarachnoid space of the temporal lobe, showing irregular morphology with uniform density. Subarachnoid hemorrhage is a possibility. Chest CT shows slight pneumonia changes.

ECG Report:

ST-T segment changes.
