# Supplementary material for: Knowledge Graph–Enhanced Deep Learning Model (H-SYSTEM) for Hypertensive Intracerebral Hemorrhage: Model Development and Validation
Source: J Med Internet Res. 2025 Jun 12;27:e66055. doi: 10.2196/66055 (PMC12203281; doi:10.2196/66055)
Supplement: Multimedia Appendix 3 [file jmir-v27-e66055-s003.docx]

**Appendix 1 Supplemental methods and results**

**Supplemental methods**

**1. Construction of H-SYSTEM**

The H-SYSTEM comprises three main modules: the key-named entity identification module, the knowledge-based semantic representation module, and the knowledge and deep learning-based semantic analysis module. These modules were meticulously designed and developed through a collaborative endeavor involving seasoned AI researchers and neurosurgeons. (Figure 4)

***1.1 Key Named Entity Identification Module***

This paper presents the construction of a BERT-IDCNN-BiLSTM-CRF model for the effective extraction of key named entities from both global and local text sources. The model leverages the BERT pre-trained language model as its foundation. Comprising four interconnected modules, namely BERT, IDCNN, BiLSTM, and CRF, the model demonstrates superior performance. The BERT module, fine-tuned for this specific task, extracts word vector representations by capturing intricate semantic information from complex medical records. These representations are then passed through the IDCNN and BiLSTM networks to optimize feature extraction. Finally, the CRF module refines the output features, ensuring accurate and reliable results.

***(1) BERT Module***

To accomplish the pre-training objective, the BERT module employs a masked language model, similar to the one utilized for a cloze test. Additionally, the BERT module incorporates a self-attentive mechanism to compute the interrelation among words within text sequences. This self-attention component empowers the BERT module to extract word embeddings for named entities, serving as feature representations across the entire sequence. The calculation of this process is presented in Equation (1)

$\mathrm{Attention}\left( Q,K,V \right)=\mathrm{softmax} \left( K^{T}Q{\sqrt{d_{k}}}^{-1} \right)V$ (1)

*Where is the input vector matrix, represents the similarity matrix calculated from the input vectors, and is the dimensionality of the input vector matrix.*

***(2) IDCNN Module***

Compared to conventional CNN models, the Inflated Dilated Convolutional Neural Network (IDCNN) minimizes information loss by modifying the expansion width between convolutional kernels. This adjustment expands the model's receptive field, enabling it to capture longer contextual information during text processing tasks. The receptive field of the inflated convolution is determined by Equation (2), which governs its perceptual range.

$F_{i+1}=\left( 2^{i+2}-1 \right)^{2}$ (2)

*The IDCNN module is composed of three convolutional layers with expansion widths of 1, 1, and 2, respectively. In total, the module undergoes four iterations. The sharing of parameters across these iterations effectively mitigates the risk of overfitting, ensuring the model's generalizability and robustness.*

***(3) BiLSTM Module***

In the context of recurrent neural networks, the Bidirectional Long Short-Term Memory (BiLSTM) architecture utilizes gating mechanisms, including input gates, output gates, and forgetting gates. These gates regulate the flow of information within the network. As a bidirectional LSTM, BiLSTM captures global contextual features from both forward and backward directions. The update equations for the states of the three gates are provided in equations (3) to (8) below:

$f_{t}=\sigma_{g}(W_{f}*[h_{t-1},u_{t}]+b_{f})$ (3)

$i_{t}=\sigma_{g}(W_{i}*[h_{t-1},u_{t}]+b_{i})$ (4)

$O_{t}=\sigma_{g}(W_{o}*\left[ h_{t-1},u_{t} \right]+b_{o})$ (5)

$C_{t}=\sigma_{h}(W_{c}*[h_{t-1},u_{t}]+b_{c})$ (6)

$C_{t}=f_{t}{*C}_{t-1}+i_{t}*C_{t}$ (7)

$h_{t}=O_{t}*\sigma_{g}\left( C_{t} \right)$ (8)

*Where is the current input, is the hidden state of the previous step, is the forgetting gate, is the input gate, is the output gate, b is the bias, represents the information remembered by the neuron at time,  is the activation function, is the hyperbolic tangent activation function, represents the information to be stored by the current neuron, and is the output of the final unit.*

***(4) CRF module***

Instead of treating each tag independently, the modeling of tag sequences is achieved through the utilization of Conditional Random Fields (CRF). CRF enables the joint modeling of tag sequences for a given input word sequence. Specifically, a probabilistic model of CRF establishes a distribution encompassing all conceivable sequences of labels, given a sequence of input words. This distribution is formulated as follows:

$P(Y\mid X)=\frac{1}{R(X)}\prod_{t=1}^{n+1} exp\left\{ W_{t}\left( Y_{t-1},Y_{t}\mid X \right) \right\}$ (9)

*where the normalization function are:*

$R(X)=\sum_{Y\in Y(x)} \prod_{t=1}^{n+1} exp\left\{ W_{t}\left( Y_{t-1},Y_{t}\mid X \right) \right\}$ (10)

$W_{t}\left( Y_{t-1},Y_{t}\mid X \right)=\sum_{k=1}^{K} w_{k}f_{k}\left( Y_{t-1},Y_{t}\mid X \right)$ (11)

*where* $w_{k}$ *is the corresponding weight and* $f_{k}$ *is the characteristic function.*

***1.2 Knowledge and deep learning based semantic analysis Module***

In order to enhance the characterization of key named entities, a Word2vec model is implemented to convert the relevant medical texts into vector representations. Additionally, this study introduces two additional components: a medical text weight scoring mechanism known as HWS and a medical text similarity measure based on domain knowledge. The objective function, also referred to as the loss function, for the model is defined as the probability of generating a central word given the background words. This function aims to optimize the model's ability to generate accurate representations of central words based on the surrounding context.

$$J(\theta)=-\frac{1}{N-2m}\sum_{i=m+1}^{N-m} \sum_{j=i\wedge j\neq i-m}^{i+m} log\left( e^{logit\left( x_{n} \right)_{w_{j}}} \right)-log\left[ \sum_{w_{k}\in vocabulary} e^{\left. logit\left( x_{n} \right)_{w_{k}} \right]} \right.$$

(12)

In this study, a similarity metric is developed to analyze and match relevant therapies based on medical performance weight scores. The Word2vec-generated medical text word vectors are leveraged to create a sentence vector representation of the key text using the following formula.

$V_{S}=\frac{\sum_{i=1}^{m} V_{i}*e^{\text{w(}i)}}{m}$ (13)

*where* $V_{S}$ *is the sentence vector representation,* $V_{i}$ *is the word vector representation of the word,* $\text{w(i)}$ *is the weight, and* $m$ *is the number of words.*

To analyze appropriate treatment measures for medical performance, we employ cosine similarity to compare significant medical texts against a weighted scale devised by medical specialists. By exploring various medical key entities, we determine the corresponding similarity thresholds that indicate significant matches. This allows us to identify and assess the relevance of potential treatment measures based on their similarity scores.

$cosim\left( V_{s1},V_{s2} \right)=\frac{V_{s1}*V_{s2}}{\left\| V_{s1} \right\|*\left\| V_{s2} \right\|}$ (14)

*where* $v_{1}，v_{2}$ *is different sentence vector representations.*

***1.3 Knowledge-based semantic representation module***

The knowledge-based semantic representation module leverages a priori knowledge to analyze the retrieved key named entities from the HICH patient's EMR. This module combines regularized logical judgments with deep learning semantic similarity matching techniques. The analytical system employs an end-to-end pipeline model that integrates deep learning approaches with prior knowledge. Natural language processing (NLP) regularization techniques and deep learning semantic similarity matching are utilized for the logical diagnosis of the entities. NLP regularization techniques facilitate the extraction of numerical data from named entities, enabling the matching of the current symptom within the range specified by the IHWSS knowledge representation. This process yields different weighted scores corresponding to the severity or significance of the symptoms.

**2. Validation of BERT-IDCNN-BiLSTM-CRF**

To evaluate the performance of the proposed key named entity recognition model, a comparison was conducted with several related models. Under the same experimental conditions, the model proposed in this paper was compared against six other models: IDCNN-CRF, BiLSTM-CRF, BiLSTM-IDCNN-CRF, BERT-CRF, BERT-IDCNN-CRF, and BERT-BiLSTM-CRF. These models were evaluated using our dataset for named entity recognition. The experiments utilized three metrics: precision rate (P), recall rate (R), and F1 value, as performance indicators for the models.

$R=\frac{\mathrm{TP}}{TP+FN}$ (1)

$P=\frac{\mathrm{TP}}{TP+FP}$ (2)

$F1=\frac{2PR}{P+R}$ (3)

*Where* $TP$ *is the number of correctly identified positive instances and* $FP$ *represents the number of incorrectly identified negative instances. denotes the number of correctly identified negative instances and* $FN$ *represents the number of incorrectly identified positive instances.*

The computer configuration used in this experiment is as follows: CPU is Intel Core i5-12400F, GPU is NVIDIA GeForce RTX 3060Ti, operating system is Windows 11, and the program runs on the PyTorch framework. For entity-level evaluation, the experimental assessment process is illustrated in Figure 1.

The evaluation of named entity recognition is based on two criteria: the correctness of entity boundaries and the accurate labeling of entity types. The main types of errors can be categorized as follows: (1) Correct text, but possibly incorrect entity types: In this case, the recognized entities correspond to the correct text, but the assigned entity types may be incorrect. (2) Incorrect text boundaries, but possibly correct main entity words and their respective entity types: Here, the recognized entities may not have the correct boundaries, but the main entity words and their associated entity types within those boundaries might be correct. By considering these two aspects, the evaluation of named entity recognition comprehensively assesses both the accuracy of the identified entities and the correctness of their respective labels.

**3. Assessment of the H-SYSTEM**

To assess the accuracy and efficiency of the H-SYSTEM, the original treatment plan for the ICH cases was set as the “gold standard” and two experienced neurosurgeons who were blinded to the processing conditions assessed the quality of outputs of the H-SYSTEM using the following scoring standard.

The whole treatment plan (full score=100 points) was divided into diagnostic measures (full score=15 points) and therapeutic measures (full score=85 points). Diagnostic measures (full score=15 points) included cerebrovascular examination (5 points), tumor stroke screening (5 points), and coagulation function examination (5 points). Therapeutic measures (full score=85 points) included surgical treatment (full score=40 points) and non-surgical treatment (full score=45 points), which was further divided into rescue measures and drug therapies. The rescue measures (full score=35 points) included vital signs monitoring (7 points), cardiopulmonary resuscitation and maintenance of vital signs (7 points), tracheal intubation (6 points), use of a ventilator (6 points), cleaning the airway (4.5 points),  and establishing venous channels (4.5 points). The drug therapies (full score=10 points) included prevention and control of epilepsy (2 points), cranial pressure reduction (6 hours after the onset) (2 points), etiology treatment (2 points), CT follow-up (6 hours after the onset) (2 points), blood pressure control (1 point), prevention and treatment of gastrointestinal bleeding (0.5 points), and hemostatic drugs (0.5 points). When the final scores differed between the two neurosurgeons, a consensus was obtained. (Table S1)

1. **Construction of the HKG**

We established the HICH knowledge graph (HKG) to augment text recognition and automated decision-making capabilities of the H-SYSTEM. Firstly, we identified the theme of the knowledge graph as “Diagnosis and Treatment of HICH”. Next, we gathered relevant information about HICH from various sources and categorize it into three parts: general medical knowledge, medical subdomain knowledge, and medical subdomain-specific knowledge.

The general medical knowledge is obtained from medical textbooks that are widely used by medical students and clinical doctors. These textbooks cover various medical fields including “Systemic Anatomy”, “Histology and Embryology”, “Biochemistry and Molecular Biology”, “Physiology”, “Medical Immunology”, “Pathology”, “Pathophysiology”, “Pharmacology”, “Diagnostics” and “Medical Imaging” among others.

The medical subdomain knowledge refers to the specialized knowledge related to neurosurgery. This includes a range of books that are commonly used by neurosurgeons for study and work. Some of these books include “Neurology”, “Youmans Neurological Surgery”, “RHOTON: Cranial Anatomy and Surgical Approaches”, “Neurosurgical Intensive Care”, “Cranial Imaging and Clinical Neuroanatomy: MRI and CT Atlas”, and “Practical Neurology”.

The medical subdomain-specific knowledge is comprised of hypertensive intracerebral hemorrhage weight system (HWS). HWS is developed based on the latest guidelines for the diagnosis and treatment of intracerebral hemorrhage, as well as the extensive clinical experience of senior neurosurgeons. This knowledge graph is designed through collaboration between neurosurgeons and AI experts. The guidelines utilized include, but are not limited to, the “American Heart Association (AHA) //American Stroke Association (ASA) Guidelines for the Management of Spontaneous Intracerebral Hemorrhage”, the “European Stroke Organisation (ESO) Guidelines on Management of Intracerebral Hemorrhage”, and the “Guidelines for the Diagnosis and Treatment of Hypertensive Intracerebral Hemorrhage by the Chinese Stroke Association (CSA) and the National Health Commission (NHC)”.

In the HKG, nodes represent entities, such as diseases, symptoms, and treatments, while edges symbolize the relationships between these entities, such as the connections between diseases and symptoms, or diseases and treatments. Based on the collected information, we have identified these nodes and edges and constructed the knowledge graph. The construction of the HKG involves three steps: First, information extraction, which extracts entities, attributes, and relationships between entities from various data sources to form an ontological knowledge representation. This process involves key techniques such as entity extraction, relationship extraction, and attribute extraction. Entity extraction, also known as Named Entity Recognition (NER), refers to the automatic identification of named entities from text data, aiming to establish "nodes" in the knowledge graph. The entity extraction task has two key aspects: finding and classifying, which involve identifying and classifying named entities. After entity extraction, a series of discrete named entities (nodes) are obtained. To derive semantic information, relationships (edges) between these entities must be extracted from related texts, connecting multiple entities or concepts into a networked knowledge structure, which is relationship extraction. Following this, rule-based extraction methods are used by defining extraction rules, such as standardized tags or manually written regular expressions, and matching these rules with the text to extract entities and their attributes. Secondly, knowledge fusion. After information extraction, the relationships between information units are flat, lacking hierarchy and logic, and there are many redundant or erroneous information fragments. Knowledge fusion combines multi-source descriptive information about the same entity or concept through methods such as entity unification, entity disambiguation, and entity linking, integrating knowledge from multiple knowledge bases into a single knowledge base. Finally, knowledge processing. The massive data obtained from information extraction and knowledge fusion result in a series of basic factual expressions, which do not equate to knowledge. To achieve a structured and networked knowledge system, quality evaluation (partially involving manual verification) is required to ensure that qualified parts are incorporated into the knowledge system, thus ensuring the quality of the knowledge base. (Figure S1)

Upon completing the construction of the knowledge graph, validation and updating are also conducted. Validation aims to verify the accuracy of the knowledge graph, while updating ensures its currency. We will regularly review and update the knowledge graph to incorporate the latest findings from medical research.

**Figure S1. Construction Process of HKG**
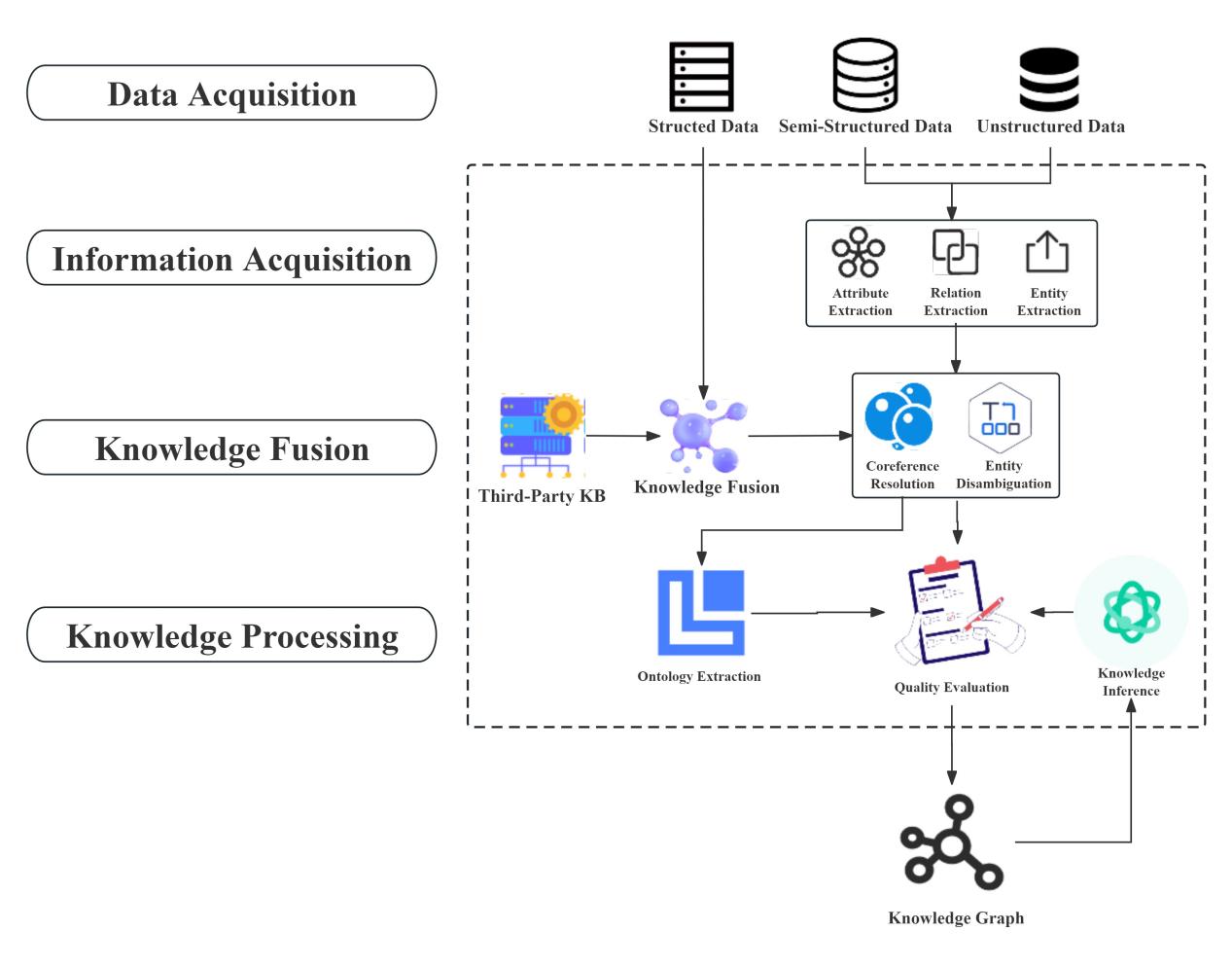


**Table S1. Scoring System Evaluation Form**

| **Item** | **Score** |
| --- | --- |
| **Total** | **100** |
| **Diagnostic measures** | **15** |
| ***Cerebrovascular examination*** | ***5*** |
| ***Tumor stroke screening*** | ***5*** |
| ***Coagulation function examination*** | ***5*** |
| **Therapeutic measures** | **85** |
| ***Surgical treatment*** | ***40*** |
| ***Non-surgical treatment*** | ***45*** |
| ***Rescue therapies*** | ***35*** |
|  | ***Vital signs monitoring (7 points)*** |
|  | ***Cardiopulmonary resuscitation and maintenance of vital signs (7 points)*** |
|  | ***Tracheal intubation (6 points)*** |
|  | ***Use of a ventilator (6 points)*** |
|  | ***Cleaning the airway (4.5 points)*** |
|  | ***Establishing venous channels (4.5 points)*** |
| ***Drug therapies*** | ***10*** |
|  | ***CT follow-up (6 hours after the onset) (2 points)*** |
|  | ***Blood pressure control (1 point)*** |
|  | ***Prevention and treatment of gastrointestinal bleeding (0.5 points)*** |
|  | ***Hemostatic drugs (0.5 points)*** |

**Supplemental results**

**1. Comprehensive effectiveness**

To test the comprehensive effectiveness of the H-SYSTEM, the same 60 cases were randomly selected from the database and provided to the ND and H-SYSTEM. For ND, the total time spent on dealing with the 60 cases was 10,679.82±1, 117.98 seconds, and the time spent on a single case was 178.00±18.63 seconds. While for the H-SYSTEM, the total time spent on dealing with the same 60 cases was 271.07±46.97 seconds, and the time for a single case was 4.52±0.79 seconds. Obviously, the speed of the H-SYSTEM in handling HICH cases was significantly higher than that of ND (p<0.001). (Table S1)

On “surgical therapy”, the accuracy, sensitivity, specificity,  and AUC were 90.00%, 90.37%, 89.63%, and 90.00% for the H-SYSTEM, and 93.87%, 94.77%, 93.23%, and 93.97% for ND. On “diagnostic measures”, the sensitivity, specificity, accuracy, and AUC were 92.53%, 95.20%, 92.77%, and 92.80%for the H-SYSTEM, and 94.77%, 89.57%, 92.07%, and 92.17% for ND. On “rescue measures”, the sensitivity, specificity, accuracy, and AUC were 90.43%, 89.43%, 89.97%, and 89.93% for the H-SYSTEM, and 89.43%, 92.20%, 90.57%, and 90.08% for ND. No statistical difference in the above sensitivity, specificity, accuracy, and AUC was found between the treatment plans of doctors and the H-SYSTEM (P<0.05).  (Table S2,)

At the same time, the number of cases handled by the H-SYSTEM and ND in a fixed time were also calculated and analyzed. Within 60 minutes, the number of cases handled by the H-SYSTEM was 460±4, which was significantly higher than the 13±5 cases handled by ND (p<0.001). Moreover, on “surgical therapy”, the accuracy, specificity, and sensitivity were 98.41%, 98.43%, and 97.43% for the H-SYSTEM, and 79.13%, 80.56%, and 75.93% for ND. On “rescue measures”, the accuracy, specificity, and sensitivity were 96.90%, 96.63%, and 97.17% for the H-SYSTEM, and 78.87%, 85.20%, and 75.00% for ND. On “diagnostic measures”, the accuracy, specificity, and sensitivity were 87.33%, 90.83%, and 85.10% for the H-SYSTEM, and 90.47% (84.16-96.77)%, 93.60% (87.03-100)%, and 88.35% (74.60-100)% for ND.  (Table S3,  Figure 3)

**Supplemental tables**

**Table S2. Comparison of time for handling 60 cases and single case among H-SYSTEM and ND**

| **Group** | **Time (60 cases)** | **Time (single case)** | **P Value** |
| --- | --- | --- | --- |
| **H-SYSTEM** | **286.80±3.82s** | **4.78s+0.69s** | **…** |
| **ND** | **10757.38±81.81s** | **178.14+19.53s** | **p<0.001** |
| When assessing 60 cases with identical conditions, including both individual case time and total time, the H-SYSTEM demonstrated significantly faster processing compared to ND | | | |

**Table S3. Comparison of efficiency for handling 60 cases between H-SYSTEM and ND**

| **Treatment plans** | **Accuracy (%) (95% CI)** | **Sensitivity (%) (95% CI)** | **Specificity (%) (95% CI)** | **AUC (%) (95% CI)** |
| --- | --- | --- | --- | --- |
| **Over all treatment plans (HD)** | **89.23 (88.10-90.56)** | **-** | **-** | **-** |
| **Over all treatment plans (ND)** | **90.86 (87.79-93.93)** | **-** | **-** | **-** |
| **P value** | **0.234** | **-** | **-** | **-** |
| **Diagnostic measures**  **(HD)** | **94,63 (90.41-98.85)** | **92.53 (86.94-98.12)** | **95.20 (90.18-100)** | **9.80 (86.37-99.23)** |
| **Diagnostic measures**  **( ND)** | **94.44 (92.05-96.83)** | **94.77 (90.43-99.11)** | **89.57 (82.92-96.22)** | **92.17 (90.16-94.17)** |
| **P value** | **0.742** | **0.423** | **0.125** | **0.643** |
| **Therapeutic measures** |  |  |  |  |
| ***Surgical therapy***  ***(HD)*** | **90.00 (85.78-94.22)** | **90.37 (82.83-97.91)** | **89.63 (87.89-91.38)** | **90.00 (85.90-94.10)** |
| ***Surgical therapy***  ***(ND)*** | **93.87 (91.42-96.30)** | **94.77 (86.44-100)** | **93.23 (85.78-100)** | **93.97 (91.94-95.99)** |
| ***P value*** | **0.123** | **0.261** | **0.234** | **0.104** |
| ***Rescue therapies***  ***(HD)*** | **89.70 (86.70-92.69)** | **90.43 (87.13-93.72)** | **89.43 (74.44-100)** | **89.93 (81.82-98.05)** |
| ***Rescue therapies***  ***(ND)*** | **89.08 (83.50-94.66)** | **89.43 (85.53-93.33)** | **92.20 (83.12-100)** | **90.80 (85.35-96.25)** |
| ***P value*** | **0.784** | **0.423** | **0.650** | **0.808** |
| Sixty cases were randomly selected from the database and distributed to both neurosurgeons and HSYSTEM for the formulation of treatment plans. The results revealed no statistically significant disparities in sensitivity, specificity, accuracy, or AUC between the treatment plans devised by the doctors and those generated by H-SYSTEM.  ND=Senior neurosurgical doctors. | | | | |

**Table S4. Comparison of quantities in 60 minutes between H-SYSTEM and ND**

| **Group** | **Quantity** | **P Value** |
| --- | --- | --- |
| **H-SYSTEM** | 460 ± 4 | ...... |
| **ND** | 13 ± 5 | p<0.001 |
| Within the same time frame, the H-SYSTEM has processed far more cases than ND. | | |


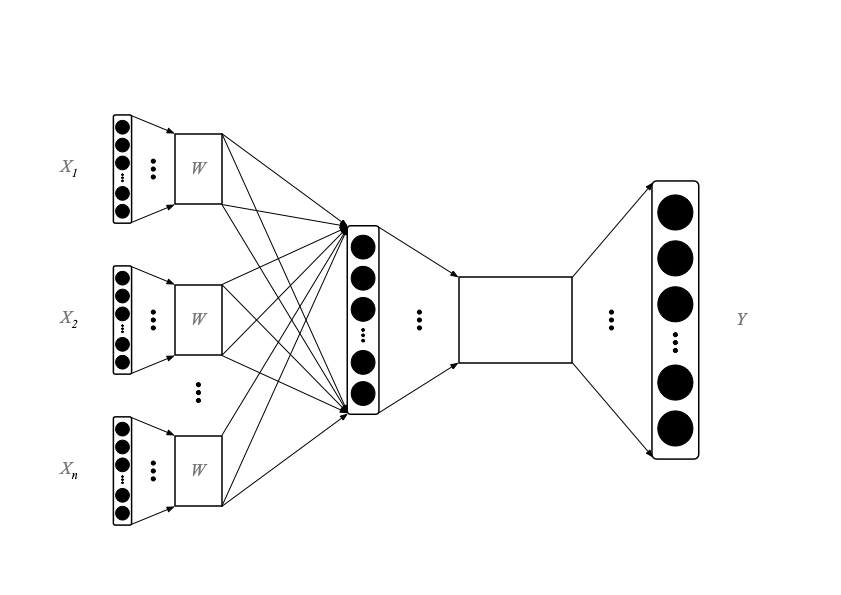


**Figure S2. Word2vec model training diagram**

**
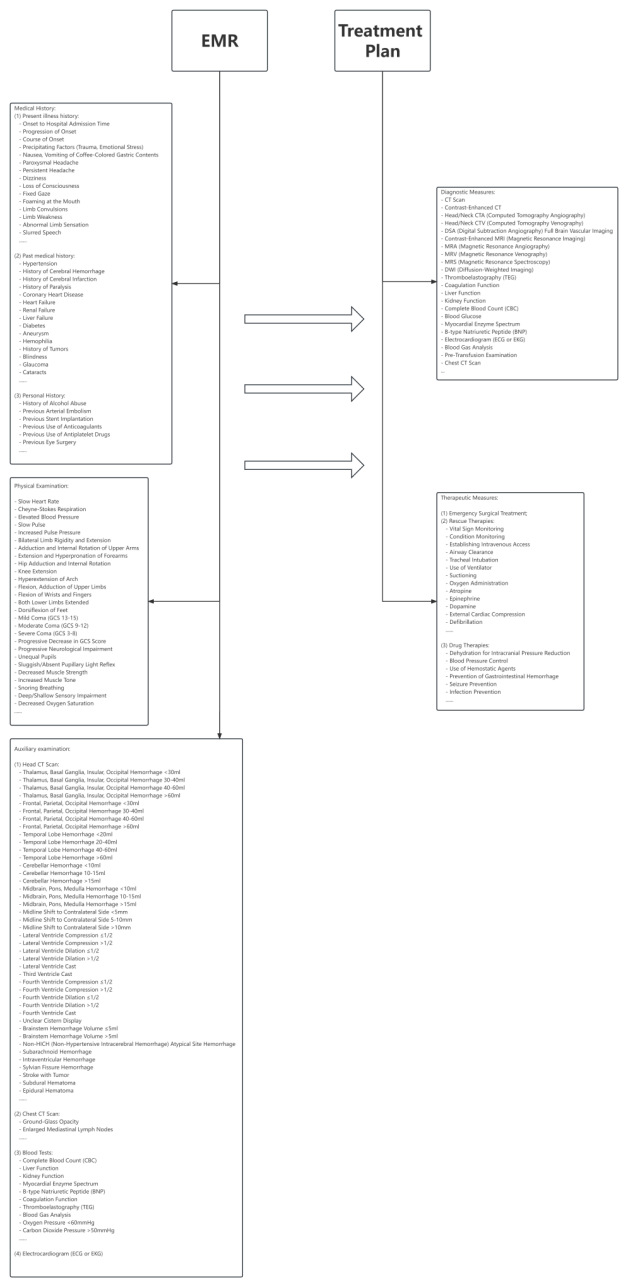
**

**Figure S2. The details of the HWS**
